# Vector control in a malaria epidemic occurring within a complex emergency situation in Burundi: a case study

**DOI:** 10.1186/1475-2875-6-93

**Published:** 2007-07-16

**Authors:** Natacha Protopopoff, Michel Van Herp, Peter Maes, Tony Reid, Dismas Baza, Umberto D'Alessandro, Wim Van Bortel, Marc Coosemans

**Affiliations:** 1 Department of Parasitology, Prince Leopold Institute of Tropical Medicine, Nationalestraat 155, B-2000 Antwerp, Belgium; 2 Medical Department, Médecins Sans Frontières Belgium, 94 rue Dupré, Brussels, Belgium; 3 Programme de Lutte contre les Maladies Transmissibles et Carentielles, Ministry of Health, Bujumbura, Burundi; 4 Department of Biomedical Sciences, Faculty of Pharmaceutical, Veterinary and Biomedical Sciences, University of Antwerp, Universiteitplein 1, B-2610, Belgium

## Abstract

**Background:**

African highlands often suffer of devastating malaria epidemics, sometimes in conjunction with complex emergencies, making their control even more difficult. In 2000, Burundian highlands experienced a large malaria outbreak at a time of civil unrest, constant insecurity and nutritional emergency. Because of suspected high resistance to the first and second line treatments, the provincial health authority and Médecins Sans Frontières (Belgium) decided to implement vector control activities in an attempt to curtail the epidemic. There are few reported interventions of this type to control malaria epidemics in complex emergency contexts. Here, decisions and actions taken to control this epidemic, their impact and the lessons learned from this experience are reported.

**Case description:**

Twenty nine hills (administrative areas) were selected in collaboration with the provincial health authorities for the vector control interventions combining indoor residual spraying with deltamethrin and insecticide-treated nets. Impact was evaluated by entomological and parasitological surveys. Almost all houses (99%) were sprayed and nets use varied between 48% and 63%. *Anopheles *indoor resting density was significantly lower in treated as compared to untreated hills, the latter taken as controls. Despite this impact on the vector, malaria prevalence was not significantly lower in treated hills except for people sleeping under a net.

**Discussion:**

Indoor spraying was feasible and resulted in high coverage despite being a logistically complex intervention in the Burundian context (scattered houses and emergency situation). However, it had little impact on the prevalence of malaria infection, possibly because it was implemented after the epidemic's peak. Nevertheless, after this outbreak the Ministry of Health improved the surveillance system, changed its policy with introduction of effective drugs and implementation of vector control to prevent new malaria epidemics.

**Conclusion:**

In the absence of effective drugs and sufficient preparedness, present study failed to demonstrate any impact of vector control activities upon the course of a short-duration malaria epidemic. However, the experience gained lead to increased preparedness and demonstrated the feasibility of vector control measures in this specific context.

## Background

Malaria epidemics are a growing problem in the African highlands with devastating effects on their immunologically naive population [1,2]. When occurring during complex emergency situations their control is even more difficult. According to WHO [3] "a complex emergency is a situation that affects large civilian populations with war or civil strife, food shortages and population displacement, resulting in excess mortality and morbidity". The approach to malaria control in the acute phases of emergencies, particularly in organized refugee camps, has been established and is based on surveillance, outbreak preparedness and case management [3,4]. However, there are a variety of situations that are much more complex where the control depends strongly on the local context.

Burundi has faced an ongoing conflict since 1993. Massive movements of the population have been recorded and according to the Office for the Coordination of Humanitarian Affairs (OCHA) more than 500,000 people were internally displaced in Burundi at the end of 2000. In addition to the civil war, Burundi faced, an increase in malaria cases in the whole country and small outbreaks were recorded in two highland provinces in the late nineties [5]. From October 2000 to March 2001, a large malaria epidemic occurred in the Burundian highlands [6], with 2.9 million registered cases over a population of 6.7 million. Between 1,000 to 8,900 probable malaria deaths were reported in three highland provinces, representing between 51% to 78% of the overall mortality [7]. This epidemic was the result of a combination of different factors including land use changes, population movements, climate variability, deteriorating health systems and malnutrition, further compounded by a high level of resistance against the main drugs chloroquine (CQ) and sulphadoxine/pyrimethamine (SP).

**Figure 1:**
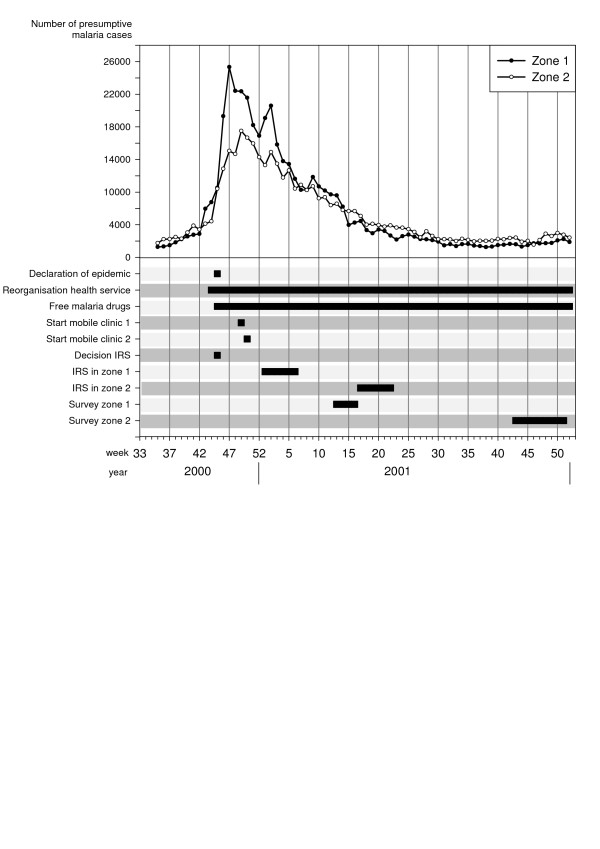
**Overview of the malaria epidemic and control interventions in the highland of Karuzi province, Burundi**. Number of presumptive malaria cases recorded separately in the health centre of Zone 1 and 2 by weeks. Decisions and actions are plot according the date of their implementation.

In Karuzi, one of the highland provinces, several actions were taken in progression to contain the increasing number of malaria cases (Figure 1). First, early November 2000, the health staff was increased, a simplified malaria treatment protocol was implemented, the hospital capacity was doubled and two mobile clinics were set up, the latter with the intention of decreasing the health facilities' workload and reaching more isolated populations. Secondly, mid-November, the Ministry of Health (MoH) declared the epidemic (Figure 1) and antimalarial drugs were provided free-of-charge. Médecins Sans Frontières Belgium (MSF-B) supplied all the public and private health facilities with CQ, SP and quinine. However, because of the suspected high CQ and SP resistance, the first and second line treatment at the time of the epidemic, the MoH in collaboration with MSF-B planned an evaluation of the resistance against these drugs. Using non efficacious drugs would not stop the epidemic and could even worsen it [8,9]. Hence, the need for an alternative strategy to control the transmission and reduce clinical malaria was required, before a new national drug policy based on the results of the resistance monitoring could be adopted.

Two additional interventions were considered. The first was indoor residual spraying (IRS), a treatment that can effectively control epidemics but usually only when implemented at an early stage of the outbreak [3]. Despite some reservations, regarding the timing of control activities, it was expected that IRS might work in this case. There is no literature describing field experiences of such an intervention to control an epidemic with conditions related to a complex emergency in the highlands. The second intervention was the use of insecticide-treated bed nets (ITN) that has been shown to reduce malaria morbidity and mortality where malaria is stable [10-12], though there is little documented evidence for the control or prevention of epidemics [13]. The malaria vectors in the Burundian highlands, *Anopheles funestus *and *Anopheles gambiae *s.l., are highly endophilic and endophagic [14-16] so that IRS or ITN or both combined had the potential of controlling the epidemic through their impact on the mosquito population.

The objective of this case study is to report on the decisions made and the actions taken to control the 2000/2001 epidemic in Karuzi province, by vector control and to present an evaluation of the programme and the lessons learned from this experience.

## Case description

### Study area

Karuzi is a poor highland province in North-East Burundi with a population of 302,000 people at the time of the epidemic. The area is hilly with altitudes ranging between 1,450 to 2,000 metres. The valleys are fertile and humid, offering breeding sites for *An. gambiae *and *An. funestus*. The annual rainfall ranges between 800 and 1,300 mm, generally between October and April. The highest mean temperatures occur between August and September (19°–20°C). The basic administrative unit is the "colline" (hill), 145 in the whole province distributed into seven communes.

### Emergency context

In Burundi, there has been a civil war since 1993. Hundred thousands of people were internally displaced or crossed the Tanzanian border. An international economic embargo further impoverished the population. Since the beginning of the conflict, and until 2000, the complex emergency, on the background of general insecurity, was characterized by displaced people, a collapsing health system, environmental deterioration and poor housing conditions. In addition, the famine that occurred in Karuzi at the end of 2000, because of the drought and poor harvest, resulted in dramatic increase of malnourished cases. A nutritional survey in November 2000 reported that 24% of the population was acutely malnourished (MSF-B unpublished data). In Karuzi, a retrospective mortality survey from November 2000 to March 2001 reported a crude mortality rate of 1.1/10,000/day, an under-five mortality rate of 3.0/10,000/day which is far above the emergency threshold of 2.0/10,000/day [7].

MSF-B started to work in Karuzi in 1993 by opening a medical emergency programme providing assistance to the local population and supporting the public health services. By mid-October 2000, the number of malaria cases in the health centres doubled over one week, a clear sign that an epidemic was beginning. In just two weeks, malaria cases increased from 17,000 to 43,330. The epidemic peaked in December (Figure 1), with a 10-fold increase of cases reported by the health centres as compared to the previous three years. The weekly number of cases remained at around 30,000 throughout January and slowly decreased the following months to return to "normal" values in May 2001.

### Vector control interventions

**Figure 2:**
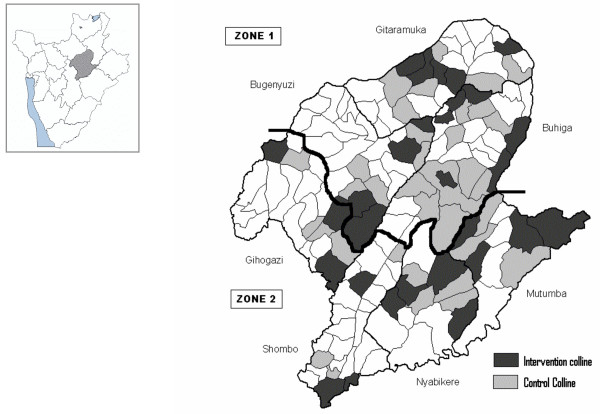
**Map of Karuzi province showing the intervention (treated hills) and control hills**. The Karuzi province is composed of "colline" (hills), represented by small polygons and regrouped in 7 communes (Buhiga, Bugenyuzi, Gitaramuka, Gihogazi, Nyabikere, Mutumba and Shombo). The dark grey polygons corresponded to the targeted hills for the vector control and light grey are the hills selected to be the control areas for the survey. The two zones (Zone 1: survey done in March-April 2001 two months after the intervention. Zone 2: survey done from October to December 2001, five months after the intervention) are separated by a thick black line.

The vector control activities were carried out in collaboration with the Transmissible and Deficiency Disease Control Programme (LMTC) and the Provincial Health Office. Despite the decision to implement vector control measures, it was impossible to cover the whole province and intervention areas had to be chosen on the basis of the malaria burden. Unfortunately, the information available was not reliable; health services were so disorganized that the patients' origin was no longer recorded and, hence, a list of the most affected areas was unavailable. Therefore, 29 hills (4–5/communes) were selected (Figure 2), regardless of more specific criteria, based on anecdotal evidence given by provincial authorities and because of insecurity in other areas.

In each commune, 14 teams (six people each) of local inhabitants were trained on IRS, following the recommended application procedure defined by Lacarin and Reed [17]. Deltamethrine 2.5 WP (K-Othrine) was applied at the target dose of 0.025 g a.i./m^2^. Each person would spray 10 houses by day. The team supervisor checked the quality of the spraying procedure and collected information on the insecticide used, the characteristics of the house and the corresponding number of people. Between December and January all health facilities, feeding centres and boarding schools were sprayed and provided with ITNs. The rest of the intervention started during the second week of January in the targeted hills of Buhiga, Bugenyuzi and Gitaramuka (Figures 1 and 2), called zone 1. The communes of Gihogazi, Mutumba, Nyabikere and Shombo were treated between April and June 2001 because of a delay in obtaining the insecticide. These communes were called zone 2 (Figures 1 and 2).

Each sprayman treated an average of 7.7 houses per day (Table 1), less than the planned target of 10 houses by day based on grouped camps or villages. Supervision was difficult due to the dispersion of the houses, the hilly environment and the absence of roads. At least once a week, some areas could not be reached because insecurity and this resulted in a delay of the supply of insecticide. Despite these problems and thanks to the good collaboration of the community, most houses (16494/16616; 99.3%) were covered by IRS (Table 1). On every intervention hill, an educational campaign for ITN was implemented before the distribution of one ITN (Permanet^® ^first generation) by household. A total of 16,781 ITNs were distributed (Table 1). In zone 2, most houses (91.8%; 95%CI: 83.8–96.6), had at least one ITN (installed or not) while this percentage was lower in zone 1 (61.2%; 95%CI: 50.0–71.6). However, the number of installed ITN was not significantly different in the two zones (zone 1: 78.8%; 95%CI: 65.3–88.9, zone 2: 69.2%; 95%CI: 57.8–79.2; *P *= 0.2).

**Table 1:** Result of the vector control activities by zone

**Indicators**	**Zone 1**	**Zone 2**
No. of inhabitants in the province	151,563	150,299
No. of inhabitants protected by IRS	32,450	36,457
% of inhabitants protected by IRS in the province	21.4%	24.3%
No. of houses in the target hills	8,853	7,763
No. of houses sprayed in the target hills (%)	8,758 (98.9%)	7,736 (99.7%)
No. of households sprayed per man/day	8.7	6.9
No. of mosquito nets distributed	8,853	7,928

### Parasitological and entomological survey

#### Survey design

Considering the emergency context no baseline survey before the vector control interventions was planned. In zone 1, a survey was carried out from 26 March to 21 April 2001 and in zone 2 from 22 October to 19 December 2001, or respectively two and five months after the end of the IRS (Figure 1). The survey includes all intervention hills. For each intervention hill, the nearest hill with the closest number of inhabitants was included as control hill (Figure 2). In each zone, the total number of houses to be selected was 85 in intervention hills and 85 in the thirty five selected control hills. The number of houses to be sampled by hill was calculated according the population density of every hill. Then from a list given by the local administration of the hill, houses were selected at random.

Daytime indoor resting mosquitoes were collected using the spray collection method [18]. After having spread white sheets on floor, the house was sprayed inside with pyrethrum, a non residual insecticide. The mosquitoes falling on the white sheets were collected and morphologically identified to species using M.T. Gillies's keys [19].

In each house, where the spray catches were done, one inhabitant was randomly selected and a rapid diagnostic test (RDT, Paracheck^®^) to detect *Plasmodium falciparum *specific antigens, was performed. People with a positive RDT were treated with oral quinine (10 mg/kg/day × 3 during seven days). Additional information on living conditions, past malaria history and treatment was also collected.

Participating individuals were informed of the objectives of the study and verbal consent was obtained. This study was a programme evaluation and was carried out with full cooperation and approval of the Burundi Ministry of Health and the Karuzi provincial authority. It was also reviewed and approved by the MSF Ethics Committee.

#### Data analysis

Data were entered into MS Excel and analysed using Epi Info version 3.3.2 (Centers for Disease Control and Prevention, Atlanta). Descriptive statistics were used to summarize demography data. Chi squared analysis was used to compare the proportions. Bivariate analyses were performed to see the relative protective effect of IRS and ITN to the outcomes using a negative binomial regression for the *Anopheles *indoor resting density and a logistic regression for the malaria prevalence (Stata intercooled version Nine). Density ratios (DR = exponential of the regression coefficient) and odds ratios (OR) are reported.

#### Results

Characteristics of the study population and selected houses are summarized in Table 2 and were similar for control and intervention hills in the same zone. In the intervention hills of zones 1 and 2 respectively, 34.1% and 44.7% of the selected persons declared having slept under a net the previous night, whereas in control areas only one person out of 170 did so. In each zone, the spray catches were done in the 170 selected households (85 in the intervention hills and 85 in the controls). In zone 1, the majority of *Anopheles *(95.2%) was *An. gambiae *s.l., the remaining being *An. funestus *while in zone 2 both species were present in almost equal proportions (*An. gambiae *s.l.: 45.1%; *An. funestus*: 54.9%). In zone 1, the protective effect of IRS against *Anopheles *in treated houses was 95% (CI 95%: 80–99) compared to control houses and adjusted for net use, in zone 2, it reached 87% (CI 95%: 31–98) (Table 3). Using a net was not followed by a significant reduction of *Anopheles *indoor resting density (Table 3). No difference in malaria infection was found between sprayed and non-sprayed hills whereas in zone 1, prevalence was lower in people sleeping under a net (Table 4). The difference in prevalence detected between the two intervention zones (zone 1: 60%, zone 2: 30%) is probably due to the natural decline of the epidemic as survey in zone 2 was carried out several months after the survey in zone 1 (Figure 1). Moreover, the proportion of persons reporting a malaria attack during the past two months was similar between control and intervention hills but was lower in October December (zone 2: 37.1%) compared to the period of March-April (zone 1: 77.1%) (Table 2).

**Table 2:** Characteristics of the study population and houses by areas (intervention hills, control hills) and by zones

	Zone 1		Zone 2	
		
	Intervention hills	Control hills	Intervention hills	Control hills
**Study population**	n = 85	n = 85	n = 85	n = 85
Median age in year (percentile 25 – percentile 75)	19 (9–38)	20 (9–40)	18 (7–32)	20 (7–37)
Proportion of women	58.8% ^a^	58.8%^a^	62.4%^a^	52.9%^a^
At least one malaria attack during the last 2 months	74.1%^a^	81.2%^a^	35.3%^b^	38.8%^b^
At least one malaria treatment the last 2 months	52.9%^a^	64.7%^a^	7.1%^b^	8.2%^b^

**Houses**	n = 85	n = 85	n = 85	n = 85
Traditional houses^1^	92.9%^a^	90.6%^a^	95.3%^a^	95.3%^a^
Roof made of thatch	56.4%^a^	69.4%^a^	49.4%^a^	41.1%^a^
Open eaves	42.4%^ac^	52.9% ^a^	29.4%^bc^	20.0%^b^
Animals inside	37.6%^a^	42.4%^a^	68.2%^b^	67.1%^b^
Houses near the marsh^2^	28.2%^a^	29.4% ^a^	47.1% ^ab^	57.6%^b^

^1 ^Walls make with mud bricks or mud, ^2 ^Houses within 500 meters

Results on the same line with identical subscript letter are not significantly different

**Table 3:** Impact of spraying and net use on *Anopheles *indoor resting density by zone using a multivariate negative binomial regression.

	Adjusted DR*	**95% CI**	**P value**
**Survey Zone 1**			
Spraying			
*Yes*	0.05	(0.01–0.20)	<0.001
*No*			
Net use			
*Yes*	0.47	(0.06–3.65)	0.470
*No*			

**Survey Zone 2**			
Spraying			
*Yes*	0.13	(0.02–0.69)	0.017
*No*			
Net use			
*Yes*	0.96	(0.14–6.58)	0.964
*No*			

* Density Ratio = exponential of the regression coefficient adjusted for net use and spraying, CI = abbreviation for Confidence Interval

**Table 4:** Impact of spraying and sleeping under net on malaria prevalence by zone using a multivariate logistic regression.

	**Prevalence % (N)**	Adjusted OR*	**95% CI**	**P value**
**Survey Zone 1**				
Spraying				
*Yes*	60.0% (85)	1.65	(0.82–3.32)	0.160
*No*	56.5% (85)			
Sleeping under net				
*Yes*	43.3% (30)	0.36	(0.15–0.88)	0.026
*No*	61.4% (140)			

**Survey Zone 2**				
Spraying				
*Yes*	28.2% (85)	0.74	(0.34–1.61)	0.446
*No*	34.1% (85)			
Sleeping under net				
*Yes*	29.0% (38)	1.07	(0.41–2.75)	0.896
*No*	31.8% (132)			

*Odd ratio adjusted for sleeping under net and spraying

## Discussion

Despite the difficulties encountered, a vector control programme based on IRS and ITN was feasible in an open setting associated with a complex emergency situation. Excellent coverage was obtained for IRS and moderately good coverage for ITN.

Ideally un-treated sentinel houses should have been chosen to evaluate the mass effect of IRS on the vector population. In present study, vector density was estimated in treated houses providing an evaluation of the treatment status of the houses. However the endophillic behaviour of *Anopheles *is very pronounced in the highlands of Burundi [15] probably restricting the resting sites in houses or shelters where the average temperatures are 3 to 5°C above the outside temperatures [16,20]. Furthermore, more than 99% of the households were sprayed including the cattle sheds and separate kitchens. It can then be assumed that the used collection method provides also a representative picture of the vector density.

IRS reduced drastically the *Anopheles *indoor resting density, although the prevalence of malaria infection did not follow accordingly. However, sleeping under a net reduced the prevalence of 64% in zone 1 whereas no difference was seen in zone 2. The absence of impact of the ITN in zone 2 can be explained by the end of the transmission period and the natural decrease in prevalence in both intervention and control hills so that no potential protective effect of the net could be seen.

The malaria cases as reported by the health centres (Figure 1) started to decline during the vector control intervention in zone 1, which could hardly be explained by the intervention itself. In zone 2 the cases reached the pre-epidemic level before the intervention. Moreover, although observed in two different control zones, malaria attacks reported during the October-December survey was half of that observed during the March April survey. Both observations suggest that the decline of the malaria incidence was mainly natural and there is no evidence that vector control activities may have sped up the resolution of the epidemic. It was mentioned earlier that IRS is useful only if applied in a timely manner at the start of the epidemic and has little or no impact on malaria epidemics if implemented when peak is reached [3]. In Burundi, the malaria epidemic was recognized late because, after 10 years of civil war, the health services were unprepared for it. Surveillance, outbreak preparedness and responses were not well developed [6]. In addition, vector control activities were started only two months after the decision had been taken despite the availability of the expertise and equipment at the LMTC. This could be explained by an underestimation of the required time and equipment due to poor information on vector control strategies in open settings, the difficulties of establishing the areas most affected and the chronic insecurity in the province which delayed the beginning of the intervention. However, vector control activities were started because good case management could not be achieved due to presumptive poor efficacy of CQ and SP. The *in vivo *resistance tests carried out afterwards reported a failure by day 14 of 93% for CQ and 66% for SP (MSF-B, internal report). These results prompted the MoH to recommend an interim drug policy with SP as a first line drug and artemether-lumefantrine to be used during malaria epidemics. The final drug policy with amodiaquine-artesunate as first line treatment was implemented at the end of 2003 [21].

The lessons learned during the 2000 epidemic encouraged the MoH to undertake measures to improve the surveillance, the response and the prevention of future malaria outbreaks. Since 2001, a weekly collection of some infectious diseases, including malaria, has been set up in all health facilities. In January 2004, the MoH and WHO elaborated a national strategy [22] to prevent, to detect earlier and to control epidemics in Burundi. This plan included, increased epidemiological surveillance, improved case management with artemisinine-based combination treatment (ACT), the strengthening of human resources in the health facilities, the distribution of mosquito nets and focal IRS in areas most at risk. Since 2005, systematic distribution of long lasting mosquito nets to pregnant women and children under five years has been integrated within routine health services. Indeed, the target groups are provided with ITN through the first antenatal cares and measles vaccination. Furthermore, acquired experience at the provincial and national level on vector control will be useful for future activities and could, with improved epidemic preparedness, greatly reduce the risk of recurrent epidemics.

Since 2001, some highland provinces were affected by higher number of malaria cases, reaching emergency thresholds in 2002 and 2005 (MoH data). However these increases were limited in time and confined to smaller areas than the 2001 epidemic. The implementation of more systematic vector control activities could be one of the reasons for the absence of true epidemics. Furthermore the introduction of ACT in December 2003 could have reduced the malaria transmission as reported in low endemic areas [23,24]. The possible acquisition of a protective immunity as observed in the Kenyan highlands population [25] could even play a more important role to explain the absence of epidemics. In Karuzi, from 2002 to 2006 a change in endemicity was observed compared to the 1998 classification of the MoH with prevalence reaching 35 to 50% in age group of two to nine years old and with a high proportion of asymptomatic carriers recorded (unpublished data).

Vector control measures based on IRS and ITN may be more appropriate for the prevention of malaria epidemics in the highlands [26,27]. One round of IRS, before the transmission period and targeted to areas near the valley marshes, could reduce the vector population, the intensity of transmission levels and the human reservoir, hence, the risk of a devastating epidemic.

## Conclusion

In the absence of effective drugs during an epidemic of malaria in the highlands of Burundi, vector control programme combining IRS and ITN was feasible despite a context of complex emergency. Vector populations were much reduced, but there is no evidence that the vector control intervention changed the natural evolution of the epidemic. This programme did, however, lead to better surveillance systems being established by the government so that future epidemics may be identified earlier. As well, the experience gained from the IRS and ITNs showed that these measures, known to be effective in preventing epidemics, could be feasibly introduced, even in the context of a complex emergency situation. The combination of improved prevention, earlier detection, and treatment with more effective drugs should help to make serious epidemics of malaria in the Burundi highlands a thing of the past.

## Authors' contributions

NP collected and analysed the data and drafted the manuscript. MC and WVB provided crucial inputs in the data analysis and the writing up of the manuscript. UD and TR improved the manuscript. MVH designed the survey and with, PM and DB facilitated the data collection and gave technical advice on the manuscript. All authors read and approved the final manuscript.
